# Implications of Epithelial–Mesenchymal Plasticity for Heterogeneity in Colorectal Cancer

**DOI:** 10.3389/fonc.2015.00013

**Published:** 2015-02-02

**Authors:** Lloyd Pereira, John M. Mariadason, Ross D. Hannan, Amardeep S. Dhillon

**Affiliations:** ^1^Research Division, Peter MacCallum Cancer Centre, Melbourne, VIC, Australia; ^2^Olivia Newton-John Cancer Research Institute, Austin Hospital, Melbourne, VIC, Australia; ^3^Sir Peter MacCallum Department of Oncology, The University of Melbourne, Melbourne, VIC, Australia; ^4^Department of Biochemistry and Molecular Biology, The University of Melbourne, Melbourne, VIC, Australia; ^5^Department of Pathology, The University of Melbourne, Melbourne, VIC, Australia

**Keywords:** CRC, epithelial–mesenchymal transition, cancer stem cell, tumor progression, subtypes, serrated

## Abstract

Colorectal cancer (CRC) is a genetically heterogeneous disease that develops and progresses through several distinct pathways characterized by genomic instability. In recent years, it has emerged that inherent plasticity in some populations of CRC cells can contribute to heterogeneity in differentiation state, metastatic potential, therapeutic response, and disease relapse. Such plasticity is thought to arise through interactions between aberrant signaling events, including persistent activation of the APC/β-catenin and KRAS/BRAF/ERK pathways, and the tumor microenvironment. Here, we highlight key concepts and evidence relating to the role of epithelial–mesenchymal plasticity as a driver of CRC progression and stratification of the disease into distinct molecular and clinicopathological subsets.

## Introduction

Colorectal cancer (CRC) has provided a paradigm for studying tumorigenesis for the past two decades (1, 2). Despite significant advances in understanding how it develops and progresses, CRC remains a major cause of cancer mortality in the developed world, due largely to its propensity to metastasize (3).

**Figure 1:**
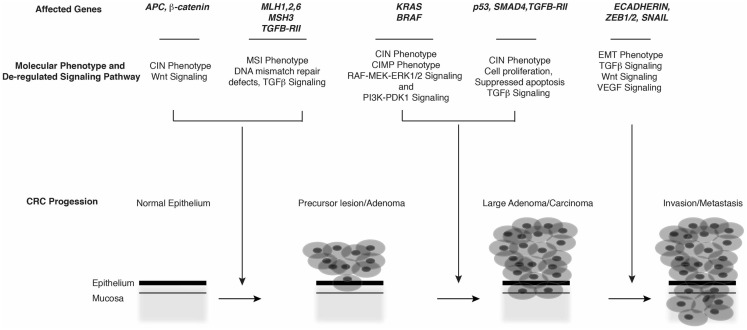
**Molecular phenotypes, genetic alterations, and major signaling pathways associated with CRC progression**.

Early models of the molecular genetics underlying sporadic and hereditary CRC suggested that it arises via clonal expansion of crypt cells bearing loss-of-function mutations in *APC* or gain-of-function *CTNNB1* mutations. Such mutations result in persistent activation of the Wnt pathway, a central regulator of stem cell compartments and cell fate along the crypt–villus axis. Aberrant Wnt signaling in CRC is characterized by localization of β-catenin to the nucleus, where it interacts with various transcription factor complexes, including TCF/LEF (4) and YAP/Tead (5), and Rel/NFκB (6). These interactions drive growth, proliferation, or stemness programs contributing to formation and progression of adenomas. Subsequent mutations in oncogenes (e.g., *KRAS*, *BRAF*) and/or tumor suppressors (e.g., *SMAD4*, *TP53*) then drive transition of adenomatous polyps to overt adenocarcinomas and subsequent metastatic disease (1, 2, 7, 8) (Figure 1).

The sequential acquisition of mutations within the adenoma-carcinoma axis, coupled with classification of disease stage/grade and histological type has provided an important paradigm to understand the “classic form” of CRC (Table 1). However, it has long been recognized that the disease is often associated with considerable heterogeneity in tumor cell phenotype, therapeutic responses, and prognoses (9–11). Indeed, comprehensive genetic and gene expression analyses have revealed variability in the genetic alterations and pathways that underlie CRC, leading to the view that the disease comprises multiple types and subtypes, which evolve through different routes (12–18). Underlying these classifications are concepts of clonal evolution, cancer stem cells (CSC), and reversible epithelial–mesenchymal transitions (EMT), each with the capacity to drive heterogeneity within CRC (6, 19–22).

**Table 1:** **Classification of CRC on the basis of the occurrence of genetic lesions, genomic stability, and histopathology**.

Genes involved	Molecular defects	Histopathology/molecular characteristics
*APC* β*-catein p53 KRAS SMAD4 TGFBR PIK3CA C-MYC*	Point mutation, aneuploidy, polyploidy, LOH, Activation of Wnt signaling pathway due to accumulated nuclear β*-catein* Deregulated TGF*β* signaling, Activation of PI3–PDK1 and RAF–MEK–ERK pathways Disruption of cell cycle regulation promoting cell survival and reduced apoptosis	**Well differentiated tumors/MSS and CIN phenotype** Familial and sporadic CRC Predominantly located in distal colon No or low mucin production Low tumor-lymphocyte reactivity
*MLH1,2,6 PMS2 MSH3, TGF-BRII*	DNA single nucleotide mismatch repair defects Alteration to micro-satellite repeat lengths Accumulation of oncogenic mutations and tumor suppressor lose Deregulated TGFβ signaling	**Poor to moderately differentiated tumors/MSI phenotype** Familial and sporadic CRC Predominantly located in distal colon Mucinous Phenotype Tumor-lymphocyte reactivity Commonly located in right colon Less aggressive/better prognosis
*BRAF MLH1*	BRAF activating point mutations Activation of RAF–MEK–ERK pathway Methylation of MLH1 and loss of MLH1 expression that is associated with mismatch repair defects	**Serrated, poor to moderately differentiated tumors/CIMP phenotype** Sporadic CRC Defective mismatch repair Commonly located in right colon Poor prognosis

*Detailed description of the characteristics used for these groupings can be found within the text and references therein*.

## EMT and Tumor Cell Plasticity During CRC Progression

That tumor heterogeneity arises through selection and expansion of different cancer cell clones bearing perturbations (e.g., mutations, epigenetic changes) conferring survival and proliferative fitness is widely accepted (1, 2, 8, 12). Heterogeneity can also arise from plasticity in tumor cell behavior, via reversible phenotypic changes driven by micro-environmental, morphogenetic, or therapeutic factors (21). These observations have in part been linked to the cancer stem cell idea, according to which a small but highly tumorigenic population of CSC having the potential to form metastases regenerates itself and progeny exhibiting a cellular hierarchy resembling normal tissue (6, 19–22).

An important source of plasticity in CRC and some other solid cancers is the EMT, which together with its reverse process, a mesenchymal–epithelial transition (MET), is essential for tissue remodeling during embryogenesis and in some pathological contexts (23, 24). Importantly, EMT–MET events also provide a framework through which solid cancers can disseminate and colonize distant sites (21, 25–31). During EMT, hallmarks of differentiated epithelia such as apico-basal polarity and cell–cell adhesions are replaced with mesenchymal traits, including rear-to-front polarity, capacity for individual cell migration, and invasion of basal lamina and blood vessels.

In addition to providing a mechanism for tumor dissemination, recent studies have identified a further pathological manifestation of EMT – endowing cancer cells with stem-like potential (32, 33) that appears critical for tumor initiation, metastasis, and relapse in CRC (6, 34, 35). The coexistence of mesenchymal and stem-like traits in cancer cells that have undergone EMT has led to the idea that they constitute “migrating CSC” from which metastases are derived (21, 36). Such cells acquire the capacity to both disseminate and successfully colonize new sites, where they are thought to redifferentiate via an MET and regain the organization of cells present in the primary tumor. This model thus provides a mechanism to explain the observation that CRC metastases often retain a similar degree of differentiation as the primary tumor.

Induction of EMT requires extensive reprograming of gene expression in response to activation of various signaling pathways. Among the best studied are the Wnt, MAPK, TGFβ, and NFκB pathways, which converge on one or more transcription factors (TFs) driving EMT in the embryo, including members of the zinc finger (SNAIL1, SNAIL2/SLUG, ZEB1, ZEB2/SIP1), bHLH (TWIST1, TWIST2), forkhead (FOXC2), or homeobox (Goosecoid, SIX1, PRRX1, PREP1) families (37–39). In CRC, multiple TFs were reported as being aberrantly expressed based on immunohistochemical and transcriptome studies, including ZEB1, ZEB2/SIP1, SNAIL1, SNAIL2/SLUG TWIST, and FOXC2 (21, 40–48). Although these TFs typically function as repressors of epithelial genes, and/or genes required for cell cycle progression, they also activate transcription in some contexts, including that of stemness-promoting genes and cell cycle inhibitors (21, 49, 50).

The effects of EMT-driven TF activation can be antagonized by several species of micro-RNA (miRNA) that in addition to repressing expression of TFs, are themselves repressed by these TFs. Such reciprocal inhibition creates self-enforcing double-negative feedback loops that dictate the epithelial–mesenchymal balance. Two such loops have been well documented to operate in colorectal and other cancer cells – ZEB/miR-200 and SNAIL/mir-34 loops (51–53). In addition to repressing EMT-TFs, the miRNAs also directly target other genes involved in regulating EMT (e.g., cytoskeletal components, Wnt pathway components) and stemness (e.g., BMI1, KLF4, SOX2), underscoring their critical functions in regulating cellular plasticity during cancer progression (26, 51, 54–57). Notably, both miR-200 family members and miR-34 are induced by the tumor suppressor p53 (58–60), whose induction of miR-34 expression was found to reduce levels of several Wnt pathway components, including LEF-1, β-catenin, WNT1, WNT3, LPR6, and AXIN2 (60–62). Reduction in Axin2 via this mechanism was also reported to promote nuclear accumulation of GSK3β, where it can phosphorylate to destabilize SNAIL1 (63).

## Association of EMT with CRC Pathology

The majority of CRCs appear moderately differentiated, with smaller subsets being well or poorly differentiated. The latter cancers are characterized by highly irregular glandular structure, aggressiveness, poor prognosis, and resistance to treatment. However, moderately differentiated tumors can also contain regions of poor differentiation, typically observed at the invasive front (21, 27, 36). Often, these cancers exhibit budding phenotype, in which individual or clusters of tumor cells detach from the tumor mass and invade into the adjacent stroma. This feature is adversely prognostic and linked with enhanced probability of metastasis to the lymph nodes, liver, or lung (36, 64, 65).

Budding tumor cells are thought to have undergone an EMT-like event, losing expression of epithelial differentiation markers while gaining the capacity to express mesenchymal and stemness markers (36, 66). In contrast to central regions of the tumor, budding cells at the invasive front also typically strongly express nuclear β-catenin, which is critical for induction of EMT programs characterized by expression of ZEB1 (42) and altered basement membrane components (67). This intra-tumoral heterogeneity in β-catenin expression is likely to arise from a range of factors, including micro-environmental signals, altered cell–cell and cell–matrix adhesion, and through cross-talk with other signaling pathways such as the ERK module (27, 36, 68, 69).

While EMT–MET events provide a framework for how differentiated CRCs may metastasize, a different model was proposed by Brabletz to account for progression of poorly differentiated cancers (21). Rather than exhibiting high plasticity, these tumors retain a poorly differentiated mesenchymal phenotype that is driven primarily by mutational events. Such cancers may have arisen prior to differentiation of stem or progenitor cells in the crypt, or from cells that have evolved from differentiated tumors but selected for mutations that render them in a stable mesenchymal-like state. A further mechanism through which selection may occur is as a result of therapies, where the relapsing tumors often displaying a mesenchymal, stem-like phenotype (21). Finally, it was suggested that the highly aggressive nature of poorly differentiated tumors may result form their propensity to metastasize through “parallel progression” routes (70), in which tumors and metastasis develop and progress concurrently.

### Association of EMT with CRC Subtypes

An important question is whether models of tumor cell plasticity involving EMT–MET events and CSC can be incorporated into current approaches for CRC subtyping. Collectively, this approach may help better define the heterogeneity observed in CRC and progress the development of targeted therapies.

### CIN, MSS/MSI, CIMP subtyping

Conventional approaches to classify colorectal tumors have centered primarily on molecular [chromosomal instability (CIN); micro-satellite stability/instability (MSS/MSI); CpG island methylator phenotype (CIMP)], and pathological (TNM grade, degree of differentiation, immunohistological markers) characteristics of the tumor (9, 71) (Table 1). These classifications recognize the various forms of global genomic and epigenetic alterations that occur during tumorigenesis (Table 1). CIN is the most common form of genomic instability in CRC that underlies the sequential deregulation of classical tumor suppressor and oncogenes including *APC*, *KRAS*, and *TP53*. In the MSI classification, genomic instability arises from the mutation or methylation-mediated silencing of genes required for DNA mismatch repair (hMLH1, hMSH2, hMSH6, and hPMS2) and based on the level of MSI, CRCs can be classified as MSI-high (MSI-H), MSI-low, or MSS. MSI tumors have a lower frequency of mutations in *KRAS* and *TP53* compared to MSS cancers, and a higher frequency of mutations in genes harboring repetitive elements in their coding sequence such as *TGFBR2* (72). Recent work indicates that as a result of this loss of TGFβRII function, MSI tumor cells lines fail to undergo EMT in response to TGFβ, which may contribute to their better prognosis (73). In the CIMP classification, tumors harbor aberrant DNA methylation patterns that result in the global epigenetic silencing of genes. Each of these pathways serves as an important classifier of disease progression and response to therapy (Table 1).

### Intrinsic EMT-associated CRC subtypes

**Figure 2:**
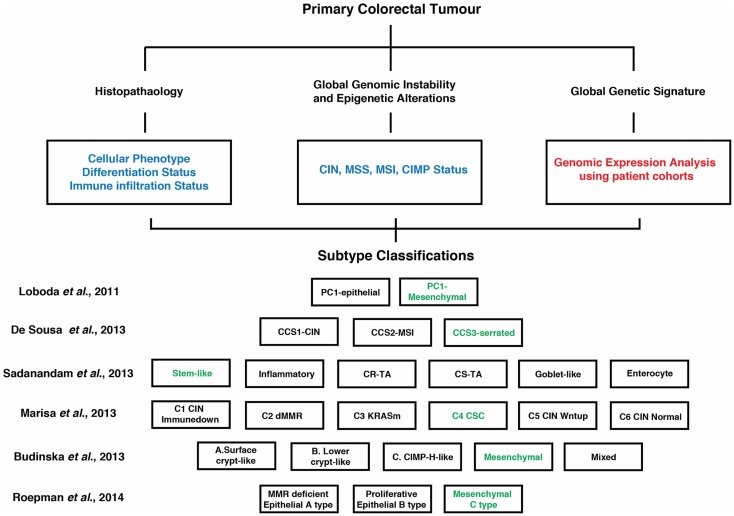
**Overview of suggested contemporary subtype classification of CRC**. Genomic and expression analyses involving large patient cohorts (highlighted in red) combined with existing mutational, clinical, and pathological classifiers (highlighted in blue) have identified several distinct molecularly defined CRC subtypes as indicated by the various studies. Each of these subtypes is driven by unique and/or overlapping signaling pathways (see Figure 1) and exhibit different prognostic and therapeutic responses. A unifying feature is a CRC subtype enriched for genes associated with a poorly differentiated, mesenchymal/invasive phenotype, and often co-enriched for genes indicative of a stem-like state (highlighted in green). A more detailed description of these subtypes and their clinical/therapeutic response can be found within the text (13, 15–17, 41, 74).

While the CIN, MSI, and CIMP are important disease sub-classifiers, it is now well-established that tumors defined by these groupings can be additionally stratified into molecularly defined subtypes. Over the past decade, genomic and expression analyses involving large patient cohorts have provided insight into the diversity within CRC. Combined with existing mutational, clinical, and pathological classifiers, these studies have identified several distinct molecularly defined CRC subtypes (e.g., stem-like, mesenchymal, immune, and epithelial/differentiated), each driven by unique and/or overlapping biological pathways and exhibiting differing prognostic and/or therapeutic response (11, 13, 15–17, 41, 74, 75) (Figure 2). A unifying feature from each of these studies was the identification of a CRC subtype significantly enriched for genes associated with a poorly differentiated, mesenchymal/invasive phenotype, and that were often co-enriched with genes indicative of a stem-like state (Figure 2).

Loboda et al. (41) defined two subsets, epithelial and mesenchymal, where the latter was linked to TGFβ signaling and low expression levels of anti-EMT miRNAs. Examination of the heterogeneity within CRC gene expression profiles also revealed a strong association between EMT gene signatures and subtyping (13). Marisa et al. (17) identified six molecular subtypes (C1–C6) from stage I–IV CRC patients, with two subtypes (C4 and C6) showing a distinct down-regulation of proliferative and upregulation of EMT/motility pathways. Subtype C4 was also characterized by a stem cell-like phenotype. Furthermore, both subtypes were distinct with regard to harboring a serrated tumor signature. Roepman et al. (74) identified three subtypes (A–C) within stages II and III CRC, with C-type tumors featuring an EMT phenotype and low proliferative activity. Two additional studies (15, 16) examined large patient-derived CRC gene expression datasets and defined CRC subtypes characterized by a mesenchymal gene signature. In the study by Sadanandam et al. (16), six subtypes were described on the basis of gene expression signatures associated with their cell of origin within the colon crypt. In this context, a stem cell subgroup was associated with expression of mesenchymal genes. De Sousa et al. (15) described three CRC subtypes (CCS1–3) and in the CCS3 grouping EMT and genes involved with migration, invasion, and TGFβ signaling were elevated. Subsequent analysis suggests that the EMT subgroups identified in both studies show strong overlap (76). Importantly, several of the above studies demonstrated that EMT signature defined tumors consistently display a worse prognosis and were least sensitive to conventional chemotherapy regimes. Thus, a mesenchymal/invasive poor differentiation signature is a defining feature of CRC subtyping and clinical response.

An important issue to emerge from the above publications is the extent to which activation of mesenchymal and stem-like programs are linked in CRC subtypes. Consistent with the role that Wnt signaling plays in regulating the fate of stem cells at the base of the crypt (8), Sadanandam et al. (16) found elevated activation of this pathway in stem-like tumors and cell lines, which co-expressed markers of intestinal and colorectal stem cells and EMT genes (34). However, whether Wnt signaling alone is sufficient to drive stem-like/mesenchymal programs expression requires further clarification as Zhu et al. (75) suggest that the pathway is not only active in mesenchymal-type tumors but also in those exhibiting differentiated or proliferative expression signatures. Instead, they found that the context of Wnt activation differed between these cancers, with migratory/EMT subsets also enriched for VEGF signaling, whereas Wnt and Notch were active in differentiated/epithelial-type tumors. Only the proliferative group (enriched for genes involved in early colon development) showed Wnt activation alone. The notion that VEGF signaling may be important for activating EMT/migration programs in the context of Wnt signaling is also supported by the finding that genes associated with sprouting angiogenesis, a process regulated by the VEGF pathway were co-enriched in mesenchymal-type tumors identified by Marisa et al. (17).

A second pathway that appears to be critical for activation of EMT programs in mesenchymal tumors is the TGFβ pathway (77, 78). Transcriptional outputs of this pathway were significantly enriched in several studies and associated with the mesenchymal phenotype (15, 17, 41, 74). Interestingly, in one study (15), TGFβ and EMT programs appeared to be active in the absence of Wnt transcriptional signatures or activation of stem cell programs. One implication of this observation is that Wnt signaling is required for stemness programs but not necessarily required for EMT in poorly differentiated cancers. Interestingly, the CCS3 group (15) enriched for sessile-serrated adenoma (SSA) tumors comprised both differentiated and poorly differentiated tumors, suggesting that further stratification based on differentiation status may be possible.

## Sessile-Serrated Adenoma Pathway

A distinct feature of CRC that has emerged from recent studies is that groups harboring an EMT gene expression signature may display a pathology related to serrated adenoma (13, 15, 17, 76). As such, the CRC subtype displaying a serrated pathology provides an important context to examine the role of EMT events in driving CRC progression.

In the classical adenoma-carcinoma sequence, tumors are often located in the distal colon or rectum and genetically are defined by CIN. In contrast, the serrated adenoma represents an alternative pathway to tumorigenesis. Typically, the serrated adenoma is located in the proximal or right colon and is characterized by the sawtooth appearance of the crypt epithelium (79). Traditionally viewed to have limited potential to progress to a neoplastic lesion, it is now established that precursor “serrated polyp” can be subdivided into hyperplastic polyp (HP), SSA, and traditional serrated adenoma (TSA) with both the SSA and TSA having significant potential to develop into neoplastic lesions (80, 81).

It has been suggested that up to 30–35% of CRCs evolve through a serrated pathway (82–84). In addition to their distinct morphology, serrated CRCs are also distinct in the genetic background that drives their development. For example, serrated colon tumors predominately display mutations in *BRAF* and *KRAS* rather than APC. With respect to the MSI and CIN classification, serrated tumors usually lack CIN but are often MSI-H and CIMP-H (71, 85, 86). Thus, serrated tumors have been classified in three subtypes: CIMP-low/MSS/MSI-low/KRAS mutant; CIMP-H/MSI-H/BRAF mutant; CIMP-low/MSS/MSI-low/BRAF mutant (9, 87). In the context of EMT-driven cellular plasticity, it is important to note that clinically CIMP-low/MSS/MSI-low/BRAF mutant tumors confer a poor prognosis and display high tumor budding. This observation is consistent with the increased EMT potential associated with wild-type TGFβRII and active TGFβ signaling and MSI-low status. In contrast CIMP-H/MSI-H/BRAF mutant tumors have a more favorable prognosis (86, 88, 89). Here, EMT potential is reduced due to the increased incidence of mutated TGFβRII (72, 73).

## Clinical Implications and Concluding Comments

The CRC classifications outlined above may provide new opportunities for the more targeted therapeutic/clinical management of CRC disease progression. This possibility is illustrated in the studies by Sadanandam et al. (16), De Sousa et al. (15), and Roepman et al. (74). Each of these studies revealed subtype-specific responses to therapy that could potentially contribute to more effective manage of disease. In case of the study by De Sousa et al., the CCS3-serrated subtype was reported to be resistant to cetuximab therapy, suggesting that new targeted therapies would be required for this subtype (15). The identification of CCS3 specific elevated TGFβ signaling suggested that this pathway may be an avenue for targeted therapy (15). The six CRC subtypes identified in the study by Sadanandam et al. (16) also displayed subtype-specific responses to therapy. Here, three subtypes, CR-TA, CS-TA, and Goblet were suggested to not respond to FOLFIRI chemotherapy treatment and patients with this form of disease may better spared this therapy in the context of local disease. However, in the context of metastatic disease, the CR-TA and CS-TA subtypes were suggested to respond to cetuximab therapy (16). In contrast, stem cell-like-subtypes and inflammatory subtypes may respond best to FOLFIRI treatment. The specific treatment of a stem cell-like subtype is an important consideration given that such populations of cells are key drivers of the moderately differentiated phenotype that are seen in most CRCs and which due to their stem-like behavior (e.g., low proliferative index) have thus far proved highly resilient to current therapies. Collectively, these studies strongly support the idea that distinct, clinically relevant CRC subtypes can be used as a guide for subtype-specific therapy.

Tumor heterogeneity has posed a major obstacle for the successful treatment of metastatic forms of CRC and several other common cancers. The studies highlighted here have provided a substantial insight into CRC heterogeneity. The identification of various degrees of epithelial–mesenchymal plasticity, acting in concert with clonal evolution and the concept of CSC, have helped dissect the heterogeneity underlying CRC and resulted in a more detailed classification of CRC into distinct molecularly defined subtypes. These classifications will provide new opportunities for understanding CRC and the key oncogenic pathways and mechanisms required for disease progression. This new information may also be invaluable for re-focusing basic and translational/pre-clinical studies on identifying and targeting key pathways required for the malignant growth of the most aggressive subtypes.

## Author Contributions

Lloyd Pereira and Amardeep Singh Dhillon conceived and drafted the manuscript. John M. Mariadason and Ross D. Hannan provided critical intellectual input and assisted with revision of the text. All authors approved the final version to be published and agree to be accountable for all aspects of the work.

## Conflict of Interest Statement

The authors declare that the research was conducted in the absence of any commercial or financial relationships that could be construed as a potential conflict of interest.
